# Distinct iron homeostasis in C57BL/6 and Balb/c mouse strains

**DOI:** 10.14814/phy2.14441

**Published:** 2020-05-08

**Authors:** Piu Saha, Xia Xiao, Yaqi Li, Rachel M. Golonka, Ahmed A. Abokor, Beng San Yeoh, Matam Vijay‐Kumar

**Affiliations:** ^1^ Department of Physiology & Pharmacology University of Toledo College of Medicine and Life Sciences Toledo OH USA; ^2^ Center for Systems Biology Massachusetts General Hospital Harvard Medical School Boston MA USA; ^3^ Department of Nutritional Sciences The Pennsylvania State University University Park PA USA; ^4^ Department of Medical Microbiology & Immunology University of Toledo College of Medicine and Life Sciences Toledo OH USA

**Keywords:** ferroportin, hepcidin, hypoferremia of inflammation, innate immunity, labile iron pool

## Abstract

C57BL/6 (BL6) and Balb/c mice exhibit prototypical Th1‐ and Th2‐dominant immune predispositions, respectively. Iron is a proinflammatory metal ion; however, limited information is documented on the differences in iron homeostasis between BL6 and Balb/c strains. The objective of this study was to investigate the extent to which strain‐level differences in these mice dictates the regulation of iron homeostasis during physiologic and inflammatory conditions. At basal levels, Balb/c mice displayed significantly higher levels of iron in systemic circulation and tissue compared to BL6 mice. Moreover, Balb/c mice had greater iron absorption as indicated by higher gene expressions of duodenal *DcytB*, *DMT1*, *Fpn*, *SFT*, and *Heph*. Similarly, hepatic *Tf, TfR1*, *TfR2*, and *DMT1* expressions were augmented in Balb/c mice. Interestingly, there was no change in hepatic *Hamp* expression between the two strains, suggesting that the disparity in their maintenance of iron is independent of hepcidin. Additionally, the basal levels of intracellular labile iron pool in Balb/c intestinal epithelial cells, and bone marrow‐derived macrophages and neutrophils, were higher compared to BL6 mice. When mice were challenged with lipopolysaccharide, the acute inflammatory response in BL6 mice was more pronounced than in Balb/c mice, as indicated by the more rapid development of hypoferremia and upregulation of serum IL‐6 and TNF‐*α* levels in BL6 mice. In conclusion, this study underscores that iron homeostasis is distinct between BL6 and Balb/c strains under both physiologic and inflammatory conditions.

## Graphical abstract

Relative to C57BL/6 mice (BL6), Balb/c mice harbor more circulating, tissue and labile iron pool (LIP), which correlates with their generally heightened expression of key iron‐regulatory proteins like divalent metal transporter 1 (DMT1) andferroportin. Accordingly, such strain‐level differences in iron homeostasis could explain the dissimilar degree of developing pro‐inflammatory responses, followed by hypoferremia, in response to inflammatory challenge like lipopolysaccharides (LPS), which occurs more rapidly in BL6 mice than Balb/c mice.

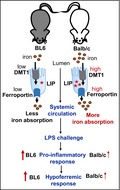

## 1 INTRODUCTION

Iron is an essential trace element required for many vital biological processes, encompassing electron and oxygen transport, cellular growth and survival, and also for inducing optimal immune responses. Therefore, iron homeostasis needs to be stringently regulated by various iron regulatory proteins that function with multi‐organ coordination throughout the absorption, transport and storage of iron. Perturbation of iron homeostasis such as in the case of iron deficiency, the most prevalent micronutrient deficiency throughout the globe, can compromise immune responses (Ahluwalia, Sun, Krause, Mastro, & Handte, 2004; Beard, 2003). Conversely, having too much iron is toxic and can increase oxidative stress and thus, exacerbate inflammation. Of note, iron overload has been shown to increase the susceptibility to many infections, presumably due to providing an iron‐rich environment for promoting the growth of pathogenic bacteria (Oppenheimer, 2001).

To restrict iron availability, the host generally responds to inflammation and/or infection by entering a state of iron deficiency known as “hypoferremia of inflammation”. The development of hypoferremia is primarily driven by hepcidin, whose expression in the liver is upregulated in response to proinflammatory cytokines, e.g., tumor necrosis factor (TNF)‐*α* and interleukin (IL)‐6 (Nemeth et al., 2004). In turn, hepcidin binds to ferroportin (Fpn; a transmembrane iron transporter expressed on duodenal enterocytes, hepatocytes and macrophages) and promotes its internalization and degradation (Coffey & Ganz, 2017). Loss of ferroportin prevents further iron absorption from the intestines and withholds iron in the liver and in iron‐scavenging cells such as macrophages, which collectively leads to hypoferremia (Coffey & Ganz, 2017). While the role of hypoferremia is well‐appreciated as a form of nutritional immunity, not much is known on whether this response could vary according to differences in iron homeostasis prior to inflammation.

C57BL/6 (BL6) and Balb/c mice are the most widely used inbred laboratory mouse strains with prototypic Th1‐ and Th2‐biased immune responses, respectively. Many studies have been performed to characterize the distinct immune response patterns between BL6 and Balb/c strains. Accordingly, BL6 mice are more predisposed to develop a Th1‐biased proinflammatory response, characterized by high interferon‐*γ* (IFN‐*γ*) and low IL‐4, whereas Balb/c mice display Th2‐biased antiinflammatory response with low IFN‐*γ* and high IL‐4, IL‐5, and IL‐10 levels (Mills, Kincaid, Alt, Heilman, & Hill, 2000). Besides their distinct T‐cell responses, the macrophages from these two strains also respond differently to various stimuli (Mills et al., 2000). For instance, macrophages from BL6 mice produce more nitric oxide than macrophages from Balb/c mice (Mills et al., 2000). In addition, when compared to BL6, Balb/c mice also exhibit higher levels of polyreactive IgA antibodies (Fransen et al., 2015).

While the immunological disparity between BL6 and Balb/c mice has been widely studied, not much is known on whether these inbred mouse strains can vary with respect to their nutritional biochemistry. Previous studies demonstrate that Balb/c mice exhibit higher levels of serum and tissue iron (Cavey et al., 2015; Hahn et al., 2009). Herein, we document that the distinct iron status between BL6 and Balb/c mice is associated with key differences in iron absorption and regulation at the tissue (i.e., duodenum, liver) and cellular (i.e., neutrophils, macrophages, enterocytes) levels. These differences in turn could explain their dissimilar pace in developing hypoferremia, in response to inflammatory challenges, which occurs more rapidly in BL6 relative to Balb/c mice. These strain‐dependent variations in iron homeostasis should be considered when generating genetically engineered knockout or transgenic mice, specifically genes related to iron homeostasis.

## 2 MATERIALS AND METHODS

### 2.1 Reagents

Duoset ELISA kits for mouse lipocalin 2 (Lcn2), interleukin (IL‐6) and tumor necrosis factor (TNF)‐*α* were purchased from R&D Systems. Iron atomic absorption (AA) standard was purchased from RICCA Chemical Company. Lipopolysaccharide (LPS; *Escherichia coli* (*E. coli*) 0128: B12), Calcein‐AM, deferoxamine (DFO), Chrome Azurol S (CAS), *RNALater* and Trizol reagents were purchased from Sigma. SYBR Green mix and qScript cDNA synthesis kits were procured from Quanta Biosciences. All other chemicals were reagent grade and procured from Sigma.

### 2.2 Mice

C57/BL6 (BL6) and Balb/c mice originally procured from Jackson Laboratory were bred and maintained (*n* = 4–5 mice/cage) with corn cob bedding and fed with a grain‐based chow diet (LabDiet #5001) at the University of Toledo. All animal experiments were approved by the Institutional Animal Care and Use Committee (IACUC) at the University of Toledo.

### 2.3 Blood collection and complete blood count

Blood was collected via heart puncture in ethylenediaminetetraacetic acid (EDTA)‐coated tubes (Greiner Bio‐one) for complete blood count (CBC) analysis. CBC was analyzed using a ProCyte DX hematology analyzer (IDEXX). The hemolysis‐free serum was collected using BD microtainer tubes (Becton Dickinson) and stored at −80°C until further use.

### 2.4 LPS challenge

Eight‐week‐old male BL6 and Balb/c mice were challenged intraperitoneally with 1.0 mg/kg of body weight of LPS from *E. coli* 0128:B12 and euthanized at indicated time points. Serum was collected for serum iron and cytokine analysis. Liver was collected and stored in *RNAlater* for gene expression analysis. In one experiment, mice were placed on an iron‐deficient diet [cat# D08090802; Research Diets Inc.] for 4 weeks before subjecting to LPS challenge as described above.

### 2.5 Isolation of bone marrow‐derived macrophages

Bone marrow‐derived macrophages (BMDM) were isolated from 5‐week‐old female mice and cultured as described in (Weischenfeldt & Porse, 2008). Briefly, bone marrow cells were isolated and cultured in six‐well plates in DMEM supplemented with 10% Fetal Bovine Serum, 1% penicillin and streptomycin, and macrophage colony stimulating factor (10 ng/ml; R&D Systems). On day 7, BMDM cultures with nearly 100% confluence were collected for labile iron pool (LIP) measurement.

### 2.6 Isolation of bone marrow‐derived neutrophils

Bone marrow‐derived neutrophils (BMDNs) were isolated from 5‐week‐old female mice using the Histopaque gradient method (Swamydas & Lionakis,^2013^). Briefly, bone marrow cells were collected by flushing the femur and tibia with 5ml of Roswell Park Memorial Institute (RPMI) 1640 medium [100U/ml penicillin/100μg/ml streptomycin, 1% FCS in phosphate‐buffered saline (PBS)], using a 25‐gauge needle, and filtering through a sterile 70‐*μ*m nylon cell strainer. Then the BMDNs were isolated by density gradient centrifugation using Histopaque. Three milliliters of Histopaque 1077 (density, 1.077g/ml) was overlaid on 3ml of Histopaque 1,119 in a 15‐ml conical tube. Then the bone marrow cell suspension (1ml PBS) was overlaid on top of the Histopaque 1077. The gradient tube was centrifuged for 30min at 800 x *g* at 25°C without brake. The neutrophils were collected at the interface of the Histopaque 1119 and Histopaque 1077 layers. The collected neutrophils were washed twice with RPMI 1640. This method yielded >95% pure and >99% viable Ly6G^+^ neutrophils, as confirmed by flow cytometry.

### 2.7 Quantification of iron parameters in serum and urine

Measurement of serum iron parameters, including total iron, total iron binding capacity (TIBC) and transferrin (Tf) saturation, was conducted as outlined previously (Walmsley, George, & Fowler, 1992; Xiao et al., 2016). Serum and urinary catalytic iron (CI) assays were analyzed as described previously (Burkitt, Milne, & Raafat, 2001). Urinary CI was normalized to creatinine measured by the creatinine kit (Randox).

### 2.8 Tissue nonheme iron assay

Liver, spleen, kidney and heart were measured for nonheme iron after acid digestion as outlined in Torrance and Bothwell (Torrance & Bothwell, 1968). Briefly, tissue samples (50 mg/ml) were digested in acid solution (3M HCl containing 10% trichloroacetic acid) and incubated at 65°C for the 20 hr. Next, the digested samples were centrifuged and 25 *μ*l of the supernatant was applied to a 96‐well microplate (Corning). Working chromogen reagent was prepared freshly on the day of assay from chromogen reagent stock (0.1% bathophenanthroline sulphonate and 1% thioglycolic acid). Next, 250 *μ*l of working chromogen reagent (1 volume of chromogen reagent stock, 5 volumes of saturated sodium acetate and 5 volumes of double deionized water) was added to the samples. After 10 min of incubation at room temperature, optical density was measured at 535 nm. A standard curve was generated using the iron atomic absorption (AA) standard and iron concentration was determined using that standard curve.

### 2.9 Quantitative RT‐PCR

Cellular and tissue RNA were extracted by using Trizol reagent (Sigma) as described in the manufacturer's protocol. mRNA (800 ng) was used to synthesize cDNA for qRT‐PCR using SYBR green (Quanta) according to the manufacturer's instruction. The following primers were used to assess gene expression: *hepcidin (Hamp)* 5′‐AGAAAGCAGGGCAGACATTG‐3′ and 5′‐CACTGGGAATTGTTACAGCATT‐3′ (Masaratana et al., 2013); *ferroportin (Fpn)* 5′‐TTGTTGTTGTGGCAGGAGAA‐3′ and 5′‐ AGCTGGTCAATCCTTCTAATGG‐3′ (Masaratana et al., 2013); *DMT1* 5′‐GGCTTTCTTATGAGCATTGCCTA‐3′ and 5′‐GGAGCACCCAGAGCAGCTTA‐3′ (Dupic et al., 2002); *DcytB* 5′‐GCAGCGGGCTCGAGTTTA‐3′ and 5′‐TTCCAGGTCCATGGCAGTCT‐3′ (Dupic et al., 2002); *Hephaestin (Heph)* 5′‐TTGTCTCATGAAGAACATTACAGCAC‐3′ and 5′‐CATATGGCAATCAAAGCAGAAGA‐3′ (Dupic et al., 2002); *SFT* 5′‐CTGTGCTCATTGAAGAGGACCTT‐3′ and 5′‐TCTGGTTGCTTTCTCAGTCACG‐3′ (Dupic et al., 2002); *Tf* 5′‐TGTAGCCTTTGTGAAACACCAGA‐3′ and 5′‐TCGGCAGGGTTCTTTCCTT‐3′ (Lyoumi et al., 2007); *TfR1* 5′‐TCATGAGGGAAATCAATGATCGTA‐3′ and 5′‐GCCCCAGAAGATATGTCGGAA‐3′ (Dupic et al., 2002); *TfR2* 5′‐GCTGGGACGGAGGTGACTT‐3′ and 5′‐GAGTTGTCCAGGCTCACGTACA‐3′ (Gao et al., 2010); *FTH* 5′‐CTCATGAGGAGAGGGAGCAT‐3′ and 5′‐GTGCACACTCCATTGCATTC‐3′ (Li et al., 2015); *FTL* 5′‐GTCCCGTGGATCTGTGTCT‐3′ and 5′‐AGGAGCTAACCGCGAAGAGA‐3′ (Li et al., 2015); *SOD2* 5′‐ CAGCCTCCCAGACCTGCCTT −3′ and 5′‐ GTGCAGGCTGAAGAGCGACC −3′ (Srinivasan et al., 2012); *Gpx* 5′‐ GGCACCACGATCCGGGACTA‐3′ and 5′‐ AAATTGGGCTCGAACCCGCC‐3′ (Srinivasan et al., 2012); *36B4* 5′–TCCAGGCTTTGGGCATCA–3′ and 5′–CTTTATTCAGCTGCACATCACTCAGA–3′ (Chassaing et al., 2012). *36B4* was used as the reference gene to normalize relative mRNA expression by Ct (2^ΔΔCt^) method. qRT‐PCR was performed in the StepOnePlus™ instrument (Life technologies).

### 2.10 Isolation of intestinal epithelial cells

Enterocytes and colonocytes from the entire small intestine and colon, respectively, were isolated for immunoblotting analysis for Fpn and for quantifying intracellular LIP as described previously (Deschemin etal.,^2016^; Deschemin & Vaulont,^2013^). This method allows for isolation of a mixed population of epithelial cells without disrupting the lamina propria that comprises the immune cell population. Briefly, small intestine (for enterocytes) and whole colon sections (for colonocytes) were incubated with 1.5mM EDTA in sterile PBS supplemented with protease inhibitor cocktail [protease inhibitor cocktail (PIC); Sigma) for 45min at 4°C, by shaking at 200rpm. Next, the residual duodenal and colonic muscle was removed, and the detached enterocytes and colonocytes were collected by centrifugation at 200 x *g* for 10min at 4°C. The cell pellet was washed once with 1X sterile PBS. For immunoblotting, the cells were resuspended immediately with lysis buffer [radioimmunoprecipitation assay (RIPA) buffer with PIC] for 30min. The samples were then centrifuged, spun at 13,500 x *g* for 10min and the supernatant was taken for protein analysis.

### 2.11 Immunoblotting

Liver tissue was homogenized in RIPA buffer with PIC and centrifuged for 15min at 4°C to remove all debris. Liver, enterocytes and RBC ghost samples prepared for ferroportin probing were denatured at 37°C, and others were denatured at 99°C for 5min. Tissue or cellular proteins were loaded with 30μg/well, fractionated using SDS‐PAGE 4%–20% gel (Bio‐Rad Laboratories, Inc.), transferred onto polyvinylidene difluoride membrane, probed with anti‐mouse ferroportin (MTP11‐A: Alpha Diagnostic International) and anti‐mouse GAPDH (Cell Signaling Technologies) or *β*‐actin as loading controls. After overnight probing with primary antibodies at 4°C, blots were then incubated with Li‐Cor secondary antibody at room temperature for 1hr. Immunoblots were developed using Li‐Cor Odyssey CLX method (Imaging Studio, Li‐Cor).

### 2.12 ELISA

Serum Lcn2, IL‐6 and TNF‐*α* were measured by Duoset ELISA kits form R&D Systems according to the manufacturer's protocol.

### 2.13 Quantification of liver thiobarbituric reactive substances (TBARS) in the liver

Liver lipid peroxidation was measured by TBARS assay as a measure of lipid peroxidation end product malondialdehyde (MDA), according to Buege and Aust (Buege & Aust, 1978).

### 2.14 Intracellular labile iron assay

BMDM, BMDN, enterocytes, and colonocytes were incubated for 15 min at 37°C and 5% CO_2_ with 0.5 μM Calcein‐AM. The cells were washed twice with PBS and treated with DFO (25 μM) for 2 hr. Following washing, cells were analyzed using a flow cytometer (Accuri c6; BD Biosciences), and the mean fluorescence intensity (MFI) was calculated using FlowJo software (Becton Dickinson). The levels of intracellular labile iron were calculated by subtracting the difference in the MFI (ΔF) before and after treatment with DFO. Percentage chelation was calculated using the formula (ΔF/control MFI) × 100.

### 2.15 Statistical analysis

All results are expressed as mean ± *SEM*. Data from two groups were analyzed using unpaired, two‐tailed *t*‐test. Data from more than two groups were compared by one‐way analysis of variance (ANOVA) followed by Tukey's multiple comparison tests to compare the mean of each column with the mean of control column. A two‐way ANOVA test was used to determine the effect of two nominal predictor variables. *p* < .05 was considered as statistically significant. All statistical analyses were performed with the GraphPad Prism 6.0 program (GraphPad Inc).

## 3 RESULTS

### 3.1 Balb/c mice maintain higher iron status than BL6 mice

**FIGURE 1:**
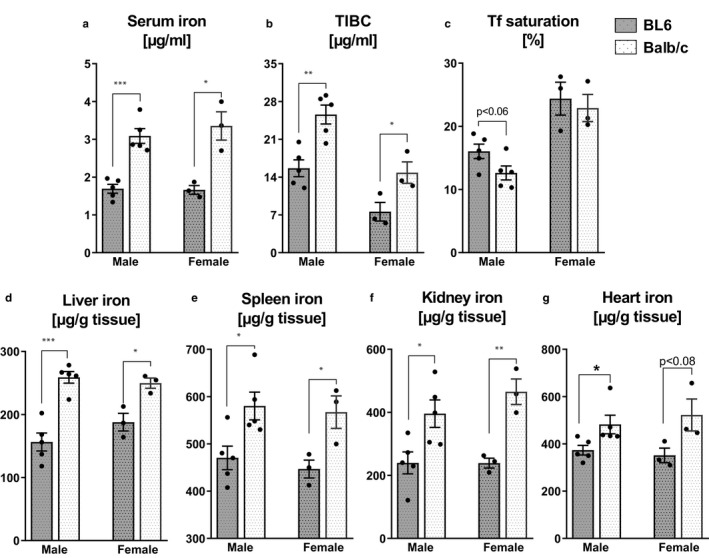
Balb/c mice display a higher iron load compared to BL6 mice. Serum and tissue samples were collected from 6‐week‐old male (*n* = 5) and female (*n* = 3) BL6 and Balb/c mice. (a–c) Circulating iron, total iron binding capacity (TIBC) and transferrin (Tf) saturation were measured in sera. (d–g) Nonheme iron levels were analyzed in the liver, spleen, kidney and heart. Data are presented as mean ± *SEM*. *p < .05, **p < .01, ***p < .001

Iron homeostasis is tightly regulated by diverse mechanisms; yet, it remains unclear whether differences in genetic background can impact iron homeostasis in two of the widely used inbred mouse strains. To compare the iron status between BL6 and Balb/c mice, we estimated their basal levels of serum total iron, total iron binding capacity (TIBC), and transferrin (Tf) saturation. In both male and female sexes, serum iron levels in Balb/c mice were ~90% more than BL6 mice (Figure 1a). Additionally, serum TIBC (Figure 1b) in Balb/c mice was also higher compared to BL6 mice, but there were no significant differences in Tf saturation (Figure 1c). Consistent with having higher serum iron levels, Balb/c mice also exhibited higher nonheme tissue iron levels, which are 62%, 21%, 65% and 29% higher in the liver, spleen, kidney and heart, respectively, when compared to BL6 mice (Figure 1d–g).

### 3.2 Balb/c mice express more proteins involved in iron uptake, transport and storage than BL6 mice

**FIGURE 2:**
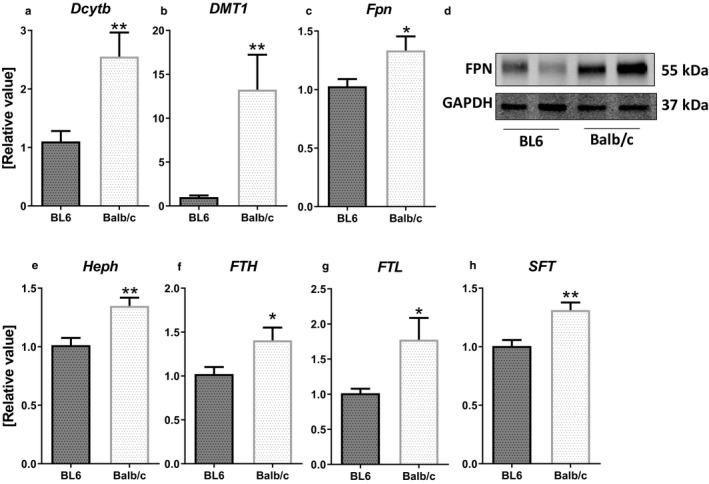
Balb/c mice exhibit higher expression of proteins involved in duodenal iron absorption. Duodenal epithelial samples were collected from 6‐week‐old male (*n* = 5) BL6 and Balb/c mice for analyzing mRNA levels of (a) *Dcytb*, (b) *DMT1*, (c) *Fpn*, (e) *Heph*, (f) *FTH*, (g) *FTL*, and (h) *SFT*. (d) FPN was also measured in protein level by immunoblotting; GAPDH was used as loading control. Data are presented as mean ± *SEM*. *p < .05, **p < .01

To explore the molecular underpinnings that could explain the higher iron status of Balb/c mice relative to BL6 mice, we next investigated the proteins involved in iron absorption, which primarily occurs in the duodenum. The duodenal transcripts for the apical iron uptake proteins, *duodenal cytochrome b* (*Dcytb*; Figure 2a) and *divalent metal transporter 1 (DMT1;* Figure 2b), were 2.5‐ and 13‐ fold higher, respectively, in Balb/c compared to BL6 mice. Moreover, the expression of the basolateral iron exporter, ferroportin (Fpn), was higher at both transcript (Figure 2c) and protein (Figure 2d) levels. In addition, basolateral ferroxidase *hephaestin* (*Heph;* Figure 2e) transcripts were 35% more abundant in Balb/c mice. The intracellular storage protein ferritin [heavy chain: *FTH* (Figure 2f) and light chain: *FTL* (Figure 2g)] and stimulator of iron transport (*SFT*; Figure 2h) were also 40%, 77% and 31% higher in Balb/c mice, respectively.

**FIGURE 3:**
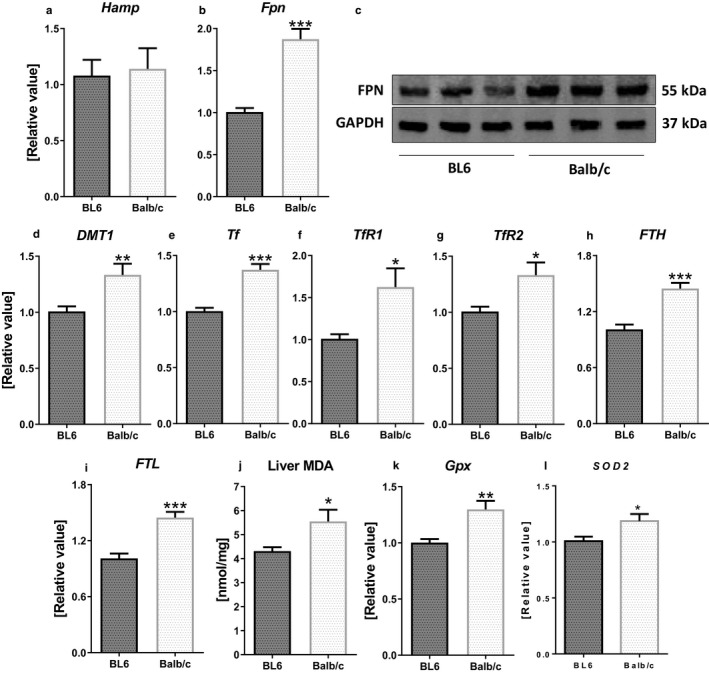
Balb/c liver expresses higher levels of iron uptake, transport and storage genes. Liver samples were collected from 6‐week‐old male (*n* = 5) BL6 and Balb/c mice for analyzing mRNA levels of (a) *Hamp*, (b) *Fpn*, (d) *DMT1*, (e) *Transferrin* (*Tf*), (f) *TfR1*, (g) *TfR2*, (h) *FTH*, (i) *FTL*. (c) Liver protein samples were used to measure FPN protein level by western blotting; GAPDH was used as loading control. J. Liver lipid peroxidation was measured as TBARS, MDA. (k–l) mRNA expression of *Gpx* (k) and *SOD2* (l) were analyzed by RT‐qPCR. Data are presented as mean ± *SEM*. *p < .05, **p < .01, ***p < .001

We next investigated the expression levels of iron regulatory proteins in the liver, which play a central role in iron homeostasis. The transcript levels for the master iron regulatory hormone, hepcidin (*Hamp*), did not differ between the two strains (Figure 3a). Nevertheless, the hepatic mRNA and protein levels for Fpn (Figure 3b and c) were significantly higher in Balb/c than BL6 mice. Likewise, Balb/c mice also expressed higher transcript levels for hepatic *DMT1*, *Tf*, *Tf receptors* (*TfR1* and *TfR2*), *FTH* and *FTL* (Figure 3d–i). Next we asked whether having more hepatic iron could increase oxidative stress in the liver. Indeed, Balb/c mice with high iron levels also displayed a significant increase in hepatic malonaldehyde (MDA; Figure 3j), the end product of lipid peroxidation. Balb/c livers also expressed more mRNA levels for the antioxidant enzymes, glutathione peroxidase (*Gpx*; Figure 3k) and superoxide dismutase 2 (*SOD2*; Figure 3l), which is perhaps a compensatory mechanism to curtail the oxidative effects of iron.

To investigate whether the distinct iron status in these mice correlates with their hematology profile, we performed complete blood count (Table S1). The results indicated that red blood cells (RBC), hemoglobin (HGB) and hematocrit (HCT) were comparable between BL6 and Balb/c mice. Interestingly, the mean corpuscular volume (MCV) was consistently lower in Balb/c mice when compared to BL6 mice with similar mean corpuscular hemoglobin (MCH), which suggests that Balb/c mice may have more condensed hemoglobin in their RBCs, as further indicated by mean corpuscular hemoglobin concentration (MCHC). It is interesting to note that RBCs from Balb/c mice have been reported to be more sensitive to oxidative hemolysis compared to RBCs from BL6 mice (Kruckeberg, 1991; Kruckeberg, Doorenbos, & Brown, 1987). A recent study demonstrated that RBCs express Fpn on their cell membranes and that increased Fpn expression reduced intracellular iron and protected RBCs from hemolysis (Zhang et al., 2018). Therefore, we envision that the sensitivity to oxidative hemolysis may be associated with distinct Fpn expression. However, we did not observe any difference in Fpn expression in RBCs from the two strains (Figure S1), suggesting that the dissimilar sensitivity of their RBCs to oxidative hemolysis could be due to factors other than Fpn.

### 3.3 Balb/c macrophages, neutrophils and epithelial cells display higher levels of intracellular LIP

Labile iron pool (LIP) (aka catalytic iron/CI) is highly reactive and can induce oxidative stress; thus, LIP needs to be stabilized by, for instance, the host innate protein Lipocalin 2 (Lcn2), which is induced in response to iron overload [14]. Lcn2 does not bind iron directly; rather, it can only stabilize LIP that is bound with the small iron chelator siderophores [14]. We did not observe any differences in either serum or urinary LIP (Figure S2a and b), along with no changes in either serum Lcn2 levels (Figure S2c) or urinary siderophore activity (Figure S2d). These results suggest that Balb/c mice could limit their systemic LIP pool, despite having a higher iron load compared to BL6 mice.

**FIGURE 4:**
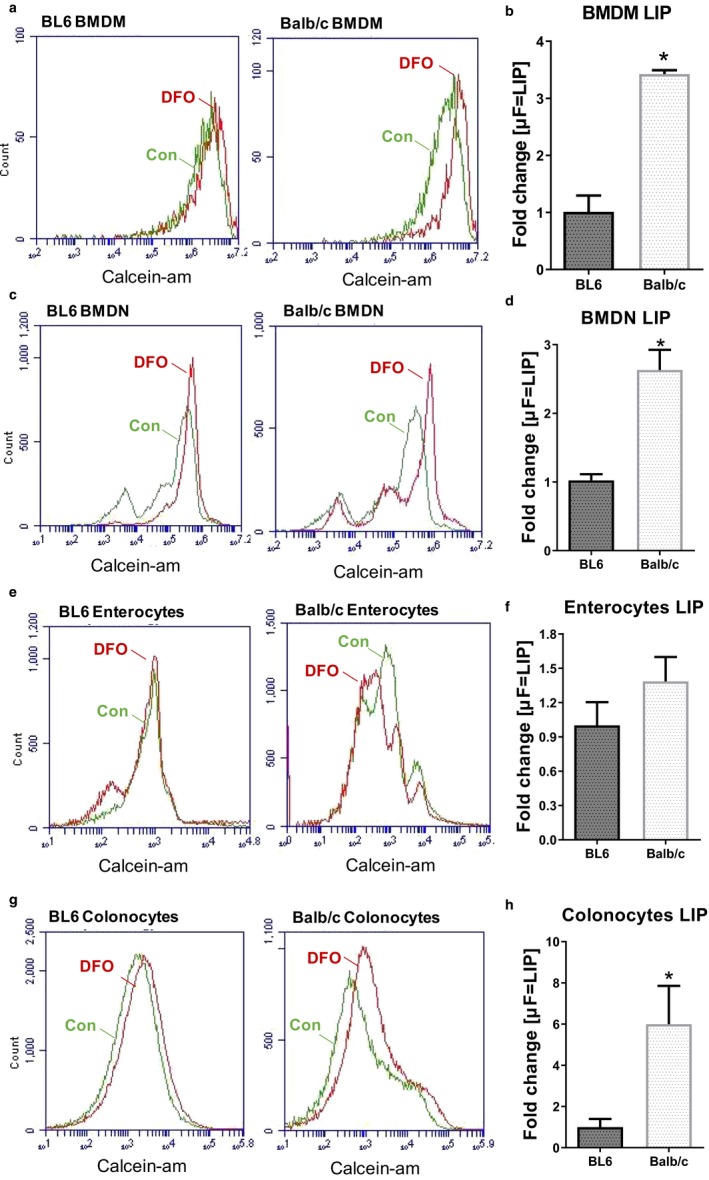
Balb/c mice have more LIP in macrophages, neutrophils and colonocytes. Bone marrow‐derived macrophages (BMDM) and neutrophils (BMDN) were isolated from 5‐week‐old female BL6 and Balb/c mice (*n* = 4). Cytosolic labile iron pool (LIP) was measured using the Calcein‐AM method by flow cytometry. Histograms and bar graphs represent the flow cytometric measurement of LIP in BL6 and Balb/c, quantified as mean fluorescence intensity (MFI), for (a–b) BMDM and (c–d) BMDN. Intestinal epithelial cells were isolated from 6‐week‐old male BL6 and Balb/c small intestine (enterocytes) and colon (colonocytes) (*n* = 3). LIP was quantified in (e–f) enterocytes and (g–h) colonocytes by flow cytometry. Data are presented as means ± *SEM*. *p < .05

We next asked whether Balb/c mice could similarly regulate their LIP in macrophages, neutrophils and epithelial cells. Macrophages, the professional phagocytes, are essential for iron homeostasis, especially for iron recycling, and in turn, the amount of LIP that modulates their immune functions (Cherayil, 2011; Wang et al., 2008). In accord with their higher systemic and storage iron levels, bone marrow‐derived macrophages (BMDM) of Balb/c mice showed 3.4‐fold higher levels of LIP (Figure 4a and b). Similar to BMDM, bone marrow‐derived neutrophils (BMDN) from Balb/c mice exhibited 2.6‐fold more LIP than BL6 BMDN (Figure 4c and d). Along with regulating immune cell responses, iron is essential for cell proliferation (Le & Richardson, 2002), where elevated iron level is beneficial in promoting tissue repair by aiding epithelial proliferation (Defrere et al., 2006). Therefore, we asked whether the elevated cellular LIP of Balb/c mice is confined to the immune cell (e.g., macrophages, neutrophils) compartments or also present in the nonimmune cell compartments (e.g., gut epithelia). While Balb/c enterocytes showed a trend of increased LIP compared to BL/6 (Figure 4e and f), Balb/c colonocytes harbored 8‐fold more intracellular LIP (Figure 4g and h).

### 3.4 Balb/c mice are more refractory to LPS‐induced hypoferremia of inflammation

**FIGURE 5:**
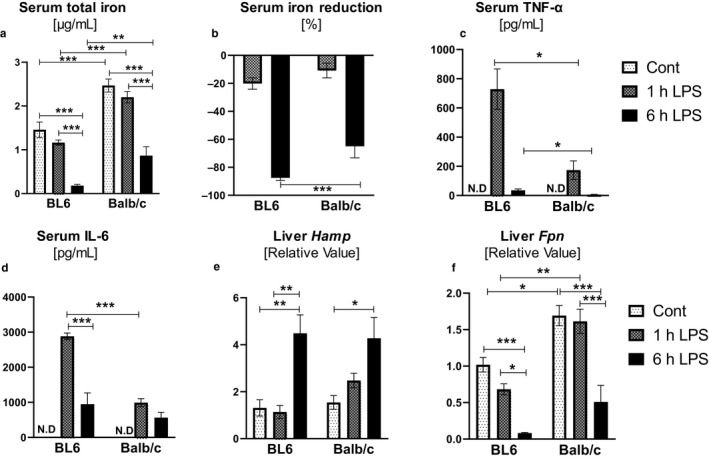
BL6 mice display more pronounced LPS‐induced hypoferremia. Six‐week‐old male BL6 and Balb/c mice were administrated *Escherichia coli* (*E. coli*) LPS (1.0 mg/kg of body weight) intraperitoneally. Serum samples were collected from LPS‐challenged mice at 1 hr and 6 hr and control groups (*n* = 4) to measure for (a) serum total iron and B. % iron reduction. (c–d) Serum TNF‐*α* and IL‐6 were quantified by ELISA. N.D. = not detected. (e–f) Liver tissue was collected for estimating mRNA levels for *Hamp* and *Fpn*. Data are presented as means ± *SEM*. Two‐way ANOVA was used to determine the statistical differences. *p < .05, **p < .01, ***p < .001

Hypoferremia of inflammation is a primitive, protective innate immune response that depletes circulating iron during infection and inflammation. As Balb/c and BL6 mice displayed a disparate profile of iron regulation, we next asked whether such differences would culminate in distinct hypoferremic responses following acute inflammation. Hence, we challenged the two strains of mice with LPS and monitored for changes in iron status at 1 and 6 hr‐time points. At 1 hr post‐LPS challenge, both strains showed a similar percent reduction in their serum iron levels (Figure 5a and b). At 6 hr, however, serum iron levels in BL6 were depleted to <0.2 μg/ml (~87% reduction), whereas Balb/c still retained approximately ~0.8 μg/ml of iron in their serum (~65% reduction) (Figure 5a and b). At 1 hr post‐LPS treatment, the cytokines involved in the hypoferremic response, TNF‐*α* and IL‐6, were 4.2‐ and 2.9‐fold higher, respectively, in BL6 relative to Balb/c sera (Figure 5c and d). Serum TNF‐*α* and IL‐6 subsided at 6 hr in both strains, but remained slightly more in BL6 compared to Balb/c mice (Figure 5c and d). Liver hepcidin expression at 1 hr post‐LPS had yet to increase in BL6 mice, but was upregulated ~1.6‐fold in Balb/c mice (Figure 5e). By 6 hr post‐LPS, both strains displayed comparable levels of upregulated *Hamp* (Figure 5e) and diminished *Fpn* (Figure 5f) expressions in the liver, *albeit* Balb/c mice still exhibited higher levels of *Fpn* expression compared to BL6 mice at this time point.

**FIGURE 6:**
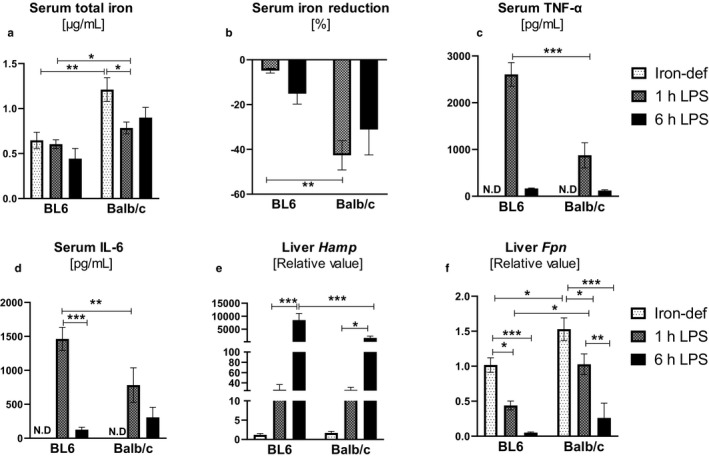
Diet‐induced hypoferremia obviates the LPS‐induced hypoferremic response in BL6 and Balb/c mice. Four‐week‐old male BL6 and Balb/c mice were maintained on iron‐deficient diet for 4 weeks and then challenged with *E. coli* LPS (1.0 mg/kg of body weight) intraperitoneally. Serum samples were collected from LPS‐challenged mice at 1 hr and 6 hr and control groups (*n* = 4) to measure for (a) serum total iron and (b) % iron reduction. (c–d) Serum TNF‐*α* and IL‐6 were quantified by ELISA. N.D. = not detected. (e–f) Liver tissue was collected for estimating mRNA levels for *Hamp* and *Fpn*. Data are presented as means ± *SEM*. Two‐way ANOVA was used to determine the statistical differences. *p < .05, **p < .01, ***p < .001

It is intriguing to note that development of hypoferremia in BL6 mice is more rapid and pronounced when compared to Balb/c mice. This disparity could likely be due, in part, to the higher levels of serum iron at baseline in Balb/c mice, which would certainly need more time for clearance, relative to BL6 mice. To address this point, we next fed both strains an iron‐deficient diet to reduce their basal iron levels prior to LPS challenge. As anticipated, 4 weeks of iron‐deficient diet feeding substantially reduced the basal serum iron levels to ~0.6 and ~1.2 μg/ml in BL6 and Balb/c mice, respectively (Figure 6a). Treating BL6 mice with LPS did not further decrease their serum iron, presumably because these mice are already in a state of hypoferremia due to the diet. Balb/c mice, which retained ~2‐fold more basal serum iron despite being fed a similar iron‐deficient diet as BL6 mice, exhibited a ~50% reduction in serum iron at 1 hr post‐LPS challenge (Figure 6a and b). Notwithstanding the blunt of hypoferremia response due to feeding an iron‐deficient diet, the upregulation of serum TNF‐*α* and IL‐6 in response to LPS remained higher in BL6 mice relative to Balb/c mice (Figure 6c and d). Furthermore, maintaining BL6 and Balb/c mice on an iron‐deficient diet augmented their upregulation of hepatic hepcidin expression, resulting in blunted *Fpn* expression to a similar extent by 6 hr post‐LPS challenge (Figure 6e and f).

Taken together, these results indicate that Balb/c mice that are predisposed to uptake and retain more iron may have slower hypoferremic responses during acute inflammation. Further studies are certainly warranted to determine whether the differences in iron status and hypoferremia responses between Balb/c and BL6 mice could be linked to their disparate susceptibility to various inflammatory or infectious diseases.

## 4 DISCUSSION

The course of inbreeding across multiple generations has segregated BL6 and Balb/c mice genetically not only by their fur color, but also by their bias toward Th1/Th2 and M1/M2 immune responses (Mills et al., 2000; Santos et al., 2006; Watanabe, Numata, Ito, Takagi, & Matsukawa, 2004). Aside from these immunological differences, BL6 and Balb/c mice also segregate from one another in their nutritional biochemistry, especially in regards to their iron management. We noted two previous studies from Cavey et al. (Cavey et al., 2015) and Hahn et al. (Hahn et al., 2009) that describe an intriguing association between iron homeostasis with the Th1/Th2 and M1/M2 bias of BL6 and Balb/c strains. Though both studies observed that Balb/c mice harbor more plasma and tissue iron than BL6 mice, they did not assess the underlying factors nor the expression of key iron regulatory genes that could explain the iron disparity in these mice. Nonetheless, the higher iron status of the M2‐biased Balb/c mice is consistent with prior studies that have shown that iron can antagonize Th1 response by inhibiting the downstream effects of IFN‐*γ* signaling (e.g., MHC class II, iNOS and TNF‐*α* expression) (Nairz et al., 2007; Oexle et al., 2003; Weiss et al., 1994) and shift the differentiation of T helper cells toward the Th2 subtype (Mencacci et al., 1997). This antagonism is bidirectional as IFN‐*γ* can promote hypoferremia of inflammation by inducing hepcidin expression in macrophages (Sow et al., 2009) and triggering iron uptake by monocytes via increasing their DMT1 (iron import) while decreasing TfR1 and Fpn1 (iron export) (Ludwiczek, Aigner, Theurl, & Weiss, 2003). Aside from favoring the Th2 bias, iron replete condition has also been shown to polarize macrophages toward the M2 subtype in in vivo and in vitro (Agoro, Taleb, Quesniaux, & Mura, 2018). M2 macrophages are noted for having high ferroportin and low ferritin expression to enhance iron release and reduce iron storage respectively (Recalcati et al., 2010). This higher degree of iron cycling in M2 macrophages is presumed to be advantageous for their role in tissue repair and angiogenesis (Recalcati et al., 2010). On the flip side, M1 macrophages generally repress ferroportin and upregulate ferritin expression to sequester and retain iron (Recalcati et al., 2010), whose redox potential is required for their bactericidal functions (Muraille, Leo, & Moser, 2014).

Iron is an essential nutrient for almost all aerobic organisms, given its necessity in many biological processes, including optimal immune responses (Nairz, Schroll, Sonnweber, & Weiss, 2010). Thus, we undertake this study to further explore whether the distinct iron levels in two widely used mouse models in biomedical research could be explained by the differences in the expression of major transporters/enzymes that maintain iron homeostasis. As previously reported (Cavey et al., 2015; Hahn et al., 2009), circulating and tissue iron levels of Balb/c mice were 2‐fold higher compared to BL6 mice; yet, the underlying factors contributing to their disparate iron status are not well‐elucidated. Consistent with their higher iron status, Balb/c mice displayed a higher iron absorption capacity on both apical (*Dcytb* and *DMT1*) and basolateral (*Fpn* and *Heph*) compartments on duodenal epithelial cells. Balb/c mice also expressed more hepatic transferrin receptors and ferritin than BL6 mice, thus suggesting that Balb/c mice can uptake and store iron at a higher rate in the liver. Despite observing the difference in hepatic iron levels, we noted that the levels of GAPDH in both strains are comparable. Though this seems to disagree with prior studies which demonstrate that iron deficiency (Quail & Yeoh, 1995) and iron loading (Sheokand et al., 2014) could upregulate hepatocyte GAPDH, we posit that this inconsistency could be because (a) our mice were in a homeostatic steady‐state and that (b) their disparity in iron status did not reach a state of either iron deficiency or excess that had been associated with a change in GAPDH expression (Quail & Yeoh, 1995; Sheokand et al., 2014). Taken together, these results affirm that Balb/c mice not only harbor more iron in their bodies within a tolerable threshold, but also express more enzymes/proteins involved in iron absorption and storage to sustain their higher iron status relative to BL6 mice.

It is intriguing that, despite both strains exhibiting a comparable level of systemic LIP, Balb/c mice harbored more LIP at the cellular level than BL6 mice. For instance, the LIP in macrophages, neutrophils and colonocytes, but not enterocytes, were greater in Balb/c compared to BL6 mice. Since macrophages and neutrophils store and utilize iron in their cellular processes, it is reasonable that their LIP level reflects the iron status of their Balb/c and BL6 background; conversely, the lack of LIP difference in enterocytes could be possibly explained by their role as a specialized epithelium that absorbs and exports iron, and thus likely to restrain iron only transiently. Although our use of calcein‐AM method to estimate cellular LIP revealed a clear difference between Balb/c and BL6 cells, this method does have its limitations that needs to be recognized. One such limitation is that calcein‐AM cannot readily permeate intracellular membranes, e.g., on lysosomes and mitochondria, *albeit* it can pass through the cell membrane via endocytosis (Tenopoulou, Kurz, Doulias, Galaris, & Brunk, 2007). Moreover, the reaction that allows calcein‐AM to fluoresce as a readout occurs optimally at pH 7 in the cytosol, but not at pH 4–5 in the lysosome (Tenopoulou et al., 2007). Hence, in principal, this method would only estimate the LIP present in the cytosolic compartment, but not the LIP present in organelles such as lysosomes. Such technical limitation may result in the underestimation of the total LIP present in Balb/c and BL6; however, the method remains to be a useful tool for assessing cellular cytosolic LIP.

As the only available putative iron exporter, hepatic Fpn plays a critical role in mobilizing iron out of the liver (Drakesmith, Nemeth, & Ganz, 2015). Increased hepatic *Fpn* expression has been found in livers with iron overload (Adams, Barbin, Khan, & Chakrabarti, 2003); therefore, to avoid iron accumulation, the higher expression of hepatic *Fpn* observed in Balb/c mice might be a compensatory response under baseline conditions. Opposingly, at the onset of inflammation, TNF‐*α* and IL‐6 initiate hypoferremia of inflammation by upregulating hepcidin expression, which in turn downregulates Fpn in liver, macrophages and duodenal enterocytes (Nemeth & Ganz, 2006). We found that BL6 mice displayed a more pronounced hypoferremic response to LPS, which correlates with higher levels of systemic TNF‐*α* and IL‐6 and an immediate blunting of hepatic *Fpn* expression at 1 hr post‐LPS, when compared to Balb/c mice. When we maintained Balb/c and BL6 mice on iron‐deficient diet before challenging them with LPS, we noted that the hypoferremia response in both strains were notably subdued. This is perhaps not surprising considering that both strains are already in a state of hypoferremia due to the diet. Nevertheless, we note that Balb/c mice still retain ~ 2‐fold more serum iron than BL6 mice, suggesting that the former may either be more resistant to iron deficiency even when exposed to a nutritional deficit diet. Intriguingly, feeding an iron‐deficient diet to both strains did not normalize the difference in their levels of serum IL‐6 and TNF‐*α* following LPS challenge. Though this outcome suggests that iron may be dispensable in Th1 response, further studies are certainly needed to clarify the cause‐effect relationship between iron homeostasis and immune responses.

The disparity between BL6 and Balb/c mice is well‐noted in their differing susceptibility to develop colitis when challenged with the chemical colitogen, dextran sodium sulfate (DSS). Specifically, Balb/c mice are more refractory to DSS‐induced colitis and thus, require treatment with twice the higher dose of DSS to induce colitis as in BL6 mice (Melgar, Karlsson, & Michaelsson, 2005). Moreover, Balb/c mice can recover from colitis much faster after DSS had been withdrawn and were symptom‐free within 2 weeks compared to BL6 mice (Melgar et al., 2005). Though the molecular underpinnings that dictate their susceptibility to colitis remains unclear, it is possible that their disease outcomes may be influenced by their disparate iron status. Of note, iron is an absolute requirement for optimal cell proliferation (Le & Richardson, 2002) and iron deficiency can impair epithelial proliferation, tissue restitution and wound healing (Wright, Richards, & Srai, 2014). The more abundant LIP in colonocytes of Balb/c mice may provide an advantage in supporting cellular proliferation and wound healing. Yet, it is worth noting that excess iron can also contribute to tumor cell proliferation (Le & Richardson, 2002). It would be interesting to determine whether the higher iron status of Balb/c could be a potential factor underlying the capability of Balb/c mice to withstand DSS‐induced colitis, but at the cost of having heightened susceptibility to develop colonic adenocarcinoma following treatment with azoxymethane and DSS, when compared to BL6 mice (Suzuki, Kohno, Sugie, Nakagama, & Tanaka, 2006).

The genetic differences between BL6 and Balb/c mice are certainly a confounding factor and this has led many studies to employ only either one of those strains. Nevertheless, many studies have sought to compare and contrast their strain‐level differences as a model to study the effects of genetic heterogeneity on various disorders (Sellers, Clifford, Treuting, & Brayton, 2012) and, more recently, on their nutritional biochemistry such as iron metabolism (Cavey et al., 2015; Hahn et al., 2009). Taken together, this study advances our knowledge on iron homeostasis in BL6 and Balb/c mice, particularly in elucidating how differential expression in key iron regulatory genes could explain their disparate iron status and hypoferremic response to acute inflammation. However, we noted several limitations in this study, e.g., the study design did not (a) adequately address whether iron status between strains differ between males and females nor (b) address cause‐effect relationship between iron status and Th1/Th2 bias. These areas of research are certainly avenues warranted for future studies. As BL6 and Balb/c mice have been reported to harbor distinct gut microbiomes (Fransen et al., 2015), it would be interesting to further investigate whether their gut microbiota has any role to play in their iron status and Th1/Th2 bias. Considering that most of the iron regulated gene knockout mice are generated on the BL6 background, such as the *Hfe*, *Hjv*, and *Hamp* deficient mice, further elucidation on iron homeostasis in BL6 and Balb/c mice may aid future studies in choosing the appropriate mouse strain for specific studies with iron metabolism and microbial pathogens.

## CONFLICT OF INTEREST

The authors have declared no conflict of interest.

## AUTHOR CONTRIBUTIONS

P.S. and X.X. designed and performed the experiments, analyzed the data and wrote the paper. Y.L., R.M.G. and A.A.A. performed experiments on animals. R.M.G. and B.S.Y. involved in discussion and editing the manuscript. M.V‐K. designed and directed the project, and co‐wrote the paper.

## Supporting information



Supplementary Material

## References

[phy214441-bib-0001] Adams, P. C. , Barbin, Y. P. , Khan, Z. A. , & Chakrabarti, S. (2003). Expression of ferroportin in hemochromatosis liver. Blood Cells, Molecules & Diseases, 31, 256–261. 10.1016/S1079-9796(03)00136-0 12972034

[phy214441-bib-0002] Agoro, R. , Taleb, M. , Quesniaux, V. F. J. , & Mura, C. (2018). Cell iron status influences macrophage polarization. PLoS ONE, 13, e0196921 10.1371/journal.pone.0196921 29771935 PMC5957380

[phy214441-bib-0003] Ahluwalia, N. , Sun, J. , Krause, D. , Mastro, A. , & Handte, G. (2004). Immune function is impaired in iron‐deficient, homebound, older women. The American Journal of Clinical Nutrition, 79, 516–521. 10.1093/ajcn/79.3.516 14985230

[phy214441-bib-0004] Beard, J. (2003). Iron deficiency alters brain development and functioning. The Journal of Nutrition, 133, 1468S–1472S. 10.1093/jn/133.5.1468S 12730445

[phy214441-bib-0005] Buege, J. A. , & Aust, S. D. (1978). Microsomal lipid peroxidation. Methods in Enzymology, 52, 302–310. 672633 10.1016/s0076-6879(78)52032-6

[phy214441-bib-0006] Burkitt, M. J. , Milne, L. , & Raafat, A. (2001). A simple, highly sensitive and improved method for the measurement of bleomycin‐detectable iron: The ‘catalytic iron index’ and its value in the assessment of iron status in haemochromatosis. Clinical Science, 100, 239–247. 11222109

[phy214441-bib-0007] Cavey, T. , Ropert, M. , de Tayrac, M. , Bardou‐Jacquet, E. , Island, M. L. , Leroyer, P. , … Loreal, O. (2015). Mouse genetic background impacts both on iron and non‐iron metals parameters and on their relationships. BioMetals, 28, 733–743. 10.1007/s10534-015-9862-8 26041486

[phy214441-bib-0008] Chassaing, B. , Srinivasan, G. , Delgado, M. A. , Young, A. N. , Gewirtz, A. T. , & Vijay‐Kumar, M. (2012). Fecal lipocalin 2, a sensitive and broadly dynamic non‐invasive biomarker for intestinal inflammation. PLoS ONE, 7, e44328 10.1371/journal.pone.0044328 22957064 PMC3434182

[phy214441-bib-0009] Cherayil, B. J. (2011). The role of iron in the immune response to bacterial infection. Immunologic Research, 50, 1–9. 10.1007/s12026-010-8199-1 21161695 PMC3085559

[phy214441-bib-0010] Coffey, R. , & Ganz, T. (2017). Iron homeostasis: An anthropocentric perspective. The Journal of Biological Chemistry, 292, 12727–12734. 28615456 10.1074/jbc.R117.781823 PMC5546013

[phy214441-bib-0011] Defrere, S. , Van Langendonckt, A. , Vaesen, S. , Jouret, M. , Gonzalez Ramos, R. , Gonzalez, D. , & Donnez, J. (2006). Iron overload enhances epithelial cell proliferation in endometriotic lesions induced in a murine model. Human Reproduction, 21, 2810–2816. 10.1093/humrep/del261 16849816

[phy214441-bib-0012] Deschemin, J. C. , Noordine, M. L. , Remot, A. , Willemetz, A. , Afif, C. , Canonne‐Hergaux, F. , … Nicolas, G. (2016). The microbiota shifts the iron sensing of intestinal cells. The FASEB Journal, 30, 252–261. 10.1096/fj.15-276840 26370847

[phy214441-bib-0013] Deschemin, J. C. , & Vaulont, S. (2013). Role of hepcidin in the setting of hypoferremia during acute inflammation. PLoS ONE, 8, e61050 10.1371/journal.pone.0061050 23637785 PMC3634066

[phy214441-bib-0014] Drakesmith, H. , Nemeth, E. , & Ganz, T. (2015). Ironing out Ferroportin. Cell Metabolism, 22, 777–787. 10.1016/j.cmet.2015.09.006 26437604 PMC4635047

[phy214441-bib-0015] Dupic, F. , Fruchon, S. , Bensaid, M. , Loreal, O. , Brissot, P. , Borot, N. , … Coppin, H. (2002). Duodenal mRNA expression of iron related genes in response to iron loading and iron deficiency in four strains of mice. Gut, 51, 648–653. 10.1136/gut.51.5.648 12377801 PMC1773425

[phy214441-bib-0016] Fransen, F. , Zagato, E. , Mazzini, E. , Fosso, B. , Manzari, C. , El Aidy, S. , … Rescigno, M. (2015). BALB/c and C57BL/6 mice differ in polyreactive IgA abundance, which impacts the generation of antigen‐specific IgA and microbiota diversity. Immunity, 43, 527–540. 10.1016/j.immuni.2015.08.011 26362264

[phy214441-bib-0017] Gao, J. , Chen, J. , De Domenico, I. , Koeller, D. M. , Harding, C. O. , Fleming, R. E. , … Enns, C. A. (2010). Hepatocyte‐targeted HFE and TFR2 control hepcidin expression in mice. Blood, 115, 3374–3381. 10.1182/blood-2009-09-245209 20177050 PMC2858491

[phy214441-bib-0018] Hahn, P. , Song, Y. , Ying, G. S. , He, X. , Beard, J. , & Dunaief, J. L. (2009). Age‐dependent and gender‐specific changes in mouse tissue iron by strain. Experimental Gerontology, 44, 594–600. 10.1016/j.exger.2009.06.006 19563877 PMC4552188

[phy214441-bib-0019] Kruckeberg, W. C. (1991). Factors influencing variable oxidative hemolysis of inbred mouse erythrocytes. Biochimica Et Biophysica Acta, 1094, 288–291. 10.1016/0167-4889(91)90088-F 1911880

[phy214441-bib-0020] Kruckeberg, W. C. , Doorenbos, D. I. , & Brown, P. O. (1987). Genetic differences in hemoglobin influence on erythrocyte oxidative stress hemolysis. Blood, 70, 909–914. 10.1182/blood.V70.4.909.909 3651609

[phy214441-bib-0021] Le, N. T. , & Richardson, D. R. (2002). The role of iron in cell cycle progression and the proliferation of neoplastic cells. Biochimica Et Biophysica Acta, 1603, 31–46. 10.1016/S0304-419X(02)00068-9 12242109

[phy214441-bib-0022] Li, W. , Garringer, H. J. , Goodwin, C. B. , Richine, B. , Acton, A. , VanDuyn, N. , … Vidal, R. (2015). Systemic and cerebral iron homeostasis in ferritin knock‐out mice. PLoS ONE, 10, e0117435 10.1371/journal.pone.0117435 25629408 PMC4309591

[phy214441-bib-0023] Ludwiczek, S. , Aigner, E. , Theurl, I. , & Weiss, G. (2003). Cytokine‐mediated regulation of iron transport in human monocytic cells. Blood, 101, 4148–4154. 10.1182/blood-2002-08-2459 12522003

[phy214441-bib-0024] Lyoumi, S. , Abitbol, M. , Andrieu, V. , Henin, D. , Robert, E. , Schmitt, C. , … Puy, H. (2007). Increased plasma transferrin, altered body iron distribution, and microcytic hypochromic anemia in ferrochelatase‐deficient mice. Blood, 109, 811–818. 10.1182/blood-2006-04-014142 17003376

[phy214441-bib-0025] Masaratana, P. , Patel, N. , Latunde‐Dada, G. O. , Vaulont, S. , Simpson, R. J. , & McKie, A. T. (2013). Regulation of iron metabolism in Hamp (‐/‐) mice in response to iron‐deficient diet. European Journal of Nutrition, 52, 135–143. 10.1007/s00394-011-0295-z 22241739

[phy214441-bib-0026] Melgar, S. , Karlsson, A. , & Michaelsson, E. (2005). Acute colitis induced by dextran sulfate sodium progresses to chronicity in C57BL/6 but not in BALB/c mice: Correlation between symptoms and inflammation. American Journal of Physiology Gastrointestinal and Liver Physiology, 288, G1328–G1338. 15637179 10.1152/ajpgi.00467.2004

[phy214441-bib-0027] Mencacci, A. , Cenci, E. , Boelaert, J. R. , Bucci, P. , Mosci, P. , Fe d'Ostiani, C. , … Romani, L. (1997). Iron overload alters innate and T helper cell responses to *Candida albicans* in mice. Journal of Infectious Diseases, 175, 1467–1476. 9180188 10.1086/516481

[phy214441-bib-0028] Mills, C. D. , Kincaid, K. , Alt, J. M. , Heilman, M. J. , & Hill, A. M. (2000). M‐1/M‐2 macrophages and the Th1/Th2 paradigm. Journal of Immunology, 164, 6166–6173. 10.4049/jimmunol.164.12.6166 10843666

[phy214441-bib-0029] Muraille, E. , Leo, O. , & Moser, M. (2014). TH1/TH2 paradigm extended: Macrophage polarization as an unappreciated pathogen‐driven escape mechanism? Frontiers in Immunology, 5, 603 10.3389/fimmu.2014.00603 25505468 PMC4244692

[phy214441-bib-0030] Nairz, M. , Schroll, A. , Sonnweber, T. , & Weiss, G. (2010). The struggle for iron ‐ a metal at the host‐pathogen interface. Cellular Microbiology, 12, 1691–1702. 10.1111/j.1462-5822.2010.01529.x 20964797

[phy214441-bib-0031] Nairz, M. , Theurl, I. , Ludwiczek, S. , Theurl, M. , Mair, S. M. , Fritsche, G. , & Weiss, G. (2007). The co‐ordinated regulation of iron homeostasis in murine macrophages limits the availability of iron for intracellular Salmonella typhimurium. Cellular Microbiology, 9, 2126–2140. 17466014 10.1111/j.1462-5822.2007.00942.x

[phy214441-bib-0032] Nemeth, E. , & Ganz, T. (2006). Regulation of iron metabolism by hepcidin. Annual Review of Nutrition, 26(1), 323–342. 10.1146/annurev.nutr.26.061505.111303 16848710

[phy214441-bib-0033] Nemeth, E. , Rivera, S. , Gabayan, V. , Keller, C. , Taudorf, S. , Pedersen, B. K. , & Ganz, T. (2004). IL‐6 mediates hypoferremia of inflammation by inducing the synthesis of the iron regulatory hormone hepcidin. Journal of Clinical Investigation, 113, 1271–1276. 10.1172/JCI200420945 15124018 PMC398432

[phy214441-bib-0034] Oexle, H. , Kaser, A. , Most, J. , Bellmann‐Weiler, R. , Werner, E. R. , Werner‐Felmayer, G. , & Weiss, G. (2003). Pathways for the regulation of interferon‐gamma‐inducible genes by iron in human monocytic cells. Journal of Leukocyte Biology, 74, 287–294. 12885946 10.1189/jlb.0802420

[phy214441-bib-0035] Oppenheimer, S. J. (2001). Iron and its relation to immunity and infectious disease. The Journal of Nutrition, 131(2), 616S–635S. 10.1093/jn/131.2.616S 11160594

[phy214441-bib-0036] Quail, E. A. , & Yeoh, G. C. (1995). The effect of iron status on glyceraldehyde 3‐phosphate dehydrogenase expression in rat liver. FEBS Letters, 359, 126–128. 10.1016/0014-5793(95)00023-3 7867783

[phy214441-bib-0037] Recalcati, S. , Locati, M. , Marini, A. , Santambrogio, P. , Zaninotto, F. , De Pizzol, M. , … Cairo, G. (2010). Differential regulation of iron homeostasis during human macrophage polarized activation. European Journal of Immunology, 40, 824–835. 10.1002/eji.200939889 20039303

[phy214441-bib-0038] Santos, J. L. , Andrade, A. A. , Dias, A. A. , Bonjardim, C. A. , Reis, L. F. , Teixeira, S. M. , & Horta, M. F. (2006). Differential sensitivity of C57BL/6 (M‐1) and BALB/c (M‐2) macrophages to the stimuli of IFN‐gamma/LPS for the production of NO: Correlation with iNOS mRNA and protein expression. Journal of Interferon & Cytokine Research, 26, 682–688. 16978073 10.1089/jir.2006.26.682

[phy214441-bib-0039] Sellers, R. S. , Clifford, C. B. , Treuting, P. M. , & Brayton, C. (2012). Immunological variation between inbred laboratory mouse strains: Points to consider in phenotyping genetically immunomodified mice. Veterinary Pathology, 49, 32–43. 22135019 10.1177/0300985811429314

[phy214441-bib-0040] Sheokand, N. , Malhotra, H. , Kumar, S. , Tillu, V. A. , Chauhan, A. S. , Raje, C. I. , & Raje, M. (2014). Moonlighting cell‐surface GAPDH recruits apotransferrin to effect iron egress from mammalian cells. Journal of Cell Science, 127(19), 4279–4291. 10.1242/jcs.154005 25074810

[phy214441-bib-0041] Sow, F. B. , Alvarez, G. R. , Gross, R. P. , Satoskar, A. R. , Schlesinger, L. S. , Zwilling, B. S. , & Lafuse, W. P. (2009). Role of STAT1, NF‐kappaB, and C/EBPbeta in the macrophage transcriptional regulation of hepcidin by mycobacterial infection and IFN‐gamma. Journal of Leukocyte Biology, 86, 1247–1258. 19652026 10.1189/jlb.1208719

[phy214441-bib-0042] Srinivasan, G. , Aitken, J. D. , Zhang, B. , Carvalho, F. A. , Chassaing, B. , Shashidharamurthy, R. , … Vijay‐Kumar, M. (2012). Lipocalin 2 deficiency dysregulates iron homeostasis and exacerbates endotoxin‐induced sepsis. Journal of Immunology, 189, 1911–1919. 10.4049/jimmunol.1200892 PMC3411903 22786765

[phy214441-bib-0043] Suzuki, R. , Kohno, H. , Sugie, S. , Nakagama, H. , & Tanaka, T. (2006). Strain differences in the susceptibility to azoxymethane and dextran sodium sulfate‐induced colon carcinogenesis in mice. Carcinogenesis, 27, 162–169. 10.1093/carcin/bgi205 16081511

[phy214441-bib-0044] Swamydas, M. , & Lionakis, M. S. (2013). Isolation, purification and labeling of mouse bone marrow neutrophils for functional studies and adoptive transfer experiments. Journal of Visualized Experiments, 77, e50586 10.3791/50586 PMC3732092 23892876

[phy214441-bib-0045] Tenopoulou, M. , Kurz, T. , Doulias, P. T. , Galaris, D. , & Brunk, U. T. (2007). Does the calcein‐AM method assay the total cellular ‘labile iron pool’ or only a fraction of it? The Biochemical Journal, 403, 261–266. 10.1042/BJ20061840 17233627 PMC1874234

[phy214441-bib-0046] Torrance, J. D. , & Bothwell, T. H. (1968). A simple technique for measuring storage iron concentrations in formalinised liver samples. The South African Journal of Medical Sciences, 33, 9–11. 5676884

[phy214441-bib-0047] Walmsley, T. A. , George, P. M. , & Fowler, R. T. (1992). Colorimetric measurement of iron in plasma samples anticoagulated with EDTA. Journal of Clinical Pathology, 45, 151–154. 10.1136/jcp.45.2.151 1541696 PMC495662

[phy214441-bib-0048] Wang, L. , Johnson, E. E. , Shi, H. N. , Walker, W. A. , Wessling‐Resnick, M. , & Cherayil, B. J. (2008). Attenuated inflammatory responses in hemochromatosis reveal a role for iron in the regulation of macrophage cytokine translation. Journal of Immunology, 181, 2723–2731. 10.4049/jimmunol.181.4.2723 PMC2561261 18684963

[phy214441-bib-0049] Watanabe, H. , Numata, K. , Ito, T. , Takagi, K. , & Matsukawa, A. (2004). Innate immune response in Th1‐ and Th2‐dominant mouse strains. Shock, 22, 460–466. 10.1097/01.shk.0000142249.08135.e9 15489639

[phy214441-bib-0050] Weischenfeldt, J. , & Porse B. . (2008). Bone marrow‐derived macrophages (BMM): Isolation and applications. *CSH protocols* 2008: pdb prot5080 10.1101/pdb.prot5080 21356739

[phy214441-bib-0051] Weiss, G. , Werner‐Felmayer, G. , Werner, E. R. , Grunewald, K. , Wachter, H. , & Hentze, M. W. (1994). Iron regulates nitric oxide synthase activity by controlling nuclear transcription. The Journal of Experimental Medicine, 180, 969–976. 10.1084/jem.180.3.969 7520477 PMC2191642

[phy214441-bib-0052] Wright, J. A. , Richards, T. , & Srai, S. K. (2014). The role of iron in the skin and cutaneous wound healing. Frontiers in Pharmacology, 5, 156 10.3389/fphar.2014.00156 25071575 PMC4091310

[phy214441-bib-0053] Xiao, X. , Yeoh, B. S. , Saha, P. , Olvera, R. A. , Singh, V. , & Vijay‐Kumar, M. (2016). Lipocalin 2 alleviates iron toxicity by facilitating hypoferremia of inflammation and limiting catalytic iron generation. BioMetals, 29(3), 451–465. 10.1007/s10534-016-9925-5 27007712 PMC4880510

[phy214441-bib-0054] Zhang, D. L. , Wu, J. , Shah, B. N. , Greutelaers, K. C. , Ghosh, M. C. , Ollivierre, H. , … Rouault, T. A. (2018). Erythrocytic ferroportin reduces intracellular iron accumulation, hemolysis, and malaria risk. Science, 359, 1520–1523. 10.1126/science.aal2022 29599243 PMC8349187

