# Supplementary material for: Distinct iron homeostasis in C57BL/6 and Balb/c mouse strains
Source: Physiol Rep. 2020 May 8;8(9):e14441. doi: 10.14814/phy2.14441 (PMC7210116; doi:10.14814/phy2.14441)
Supplement: Supplementary file 1 — Supplementary Material [file PHY2-8-e14441-s001.docx]

**Supplementary methods:**

**ELISA**

Serum Lcn2 was measured by Duoset ELISA kits form R&D Systems according to the manufacturer’s protocol.

**Urinary siderophore estimation via Chrome Azurol S (CAS) assay**

CAS liquid reagent was prepared as previously described (3). The principle of this assay is that CAS remains blue in color when complexed with iron, but turns orange when iron is chelated by urinary iron chelators/siderophores. The intensity of the orange color is directly proportional to the amount of iron chelation by iron free siderophores (3).

**Quantification of iron parameters in serum and urine**

Serum and urinary catalytic iron (CI) assays were analyzed as described previously (1). Urinary CI was normalized to creatinine measured by the creatinine kit (Randox, Crumlin, UK).

**Preparation of RBC ghosts**

RBC ghosts were prepared for Fpn analysis as described in (2, 4). Briefly, blood collected in EDTA-coated tubes (Greiner Bio-one, Monroe, NC, USA) was transferred to 1.5 ml Eppendorf tubes and centrifuged at 1,000 *g* for 10 min to remove the plasma. The cells washed with 1.0 ml of 0.172M Tris buffer (Sigma, St. Louis, MO, USA) for three times to remove buffy coat and cells were lysed with 1.0 ml of 0.011M Tris buffer, stand for 5 min and centrifuged at 14,000 *g* for 15 min. The resulting membrane fraction was washed with the 0.011M Tris buffer three times until the pellet was colorless. RBC ghost membranes were obtained by centrifugation at 14,000 *g* for 5 min, and then lysed in 5 volumes of RIPA buffer for immunoblotting. All the reagents and sample preparations were at 4ºC.

**Supplemental figure S1. Erythrocytes ferroportin expression is comparable between BL6 and Balb/c.** Enterocyte membrane ghosts were collected and purified from 6 week old male BL6 and Balb/c mice whole blood for immunoblotting analysis. Representative of ferroportin (Fpn) western blot are shown (n=2). β-actin and Ponceau staining were used as loading controls.

**Supplemental figure S2. Iron-associated indices in BL6 and Balb/c mice.** Serum and urine samples were collected from 6 week old male (n=5) BL6 and Balb/c mice. **A-D.** Serum and urinary labile iron pool (LIP), serum Lcn2 and urinary siderophores were measured. Data are presented as means ± SEM.

**Supplemental Table 1. The complete blood count of 6 week old male BL6 and Balb/c mice.** Values are mean ± SEM, n=5. ****P* < 0.0001.

|  | **BL6** | **Balb/c** | **p-Value** |
| --- | --- | --- | --- |
| **RBC [M/μL]** | 9.272 ± 0.2452 | 9.658 ± 0.112 | 0.1901 |
| **HGB [g/dL]** | 14.1 ± 0.4743 | 14.98 ± 0.2131 | 0.129 |
| **HCT [%]** | 45.88 ± 1.405 | 45.12 ± 0.6111 | 0.6332 |
| **MCV [fL]** | 49.44 ± 0.2926 | 46.7 ± 0.2775 | 0.0001*** |
| **MCH [pg]** | 15.22 ± 0.153 | 15.5 ± 0.07071 | 0.1352 |
| **MCHC [g/dL]** | 30.72 ± 0.3007 | 33.22 ± 0.1281 | < 0.0001*** |

References:

1. **Burkitt MJ, Milne L, and Raafat A**. A simple, highly sensitive and improved method for the measurement of bleomycin-detectable iron: the 'catalytic iron index' and its value in the assessment of iron status in haemochromatosis. *Clinical science* 100: 239-247, 2001.

2. **Hanahan DJ, and Ekholm JE**. The preparation of red cell ghosts (membranes). *Methods in enzymology* 31: 168-172, 1974.

3. **Xiao X, Yeoh BS, Saha P, Tian Y, Singh V, Patterson AD, and Vijay-Kumar M**. Modulation of urinary siderophores by the diet, gut microbiota and inflammation in mice. *The Journal of nutritional biochemistry* 41: 25-33, 2017.

4. **Zhang DL, Wu J, Shah BN, Greutelaers KC, Ghosh MC, Ollivierre H, Su XZ, Thuma PE, Bedu-Addo G, Mockenhaupt FP, Gordeuk VR, and Rouault TA**. Erythrocytic ferroportin reduces intracellular iron accumulation, hemolysis, and malaria risk. *Science* 359: 1520-1523, 2018.
